# WDR79 mediates the proliferation of non‐small cell lung cancer cells by regulating the stability of UHRF1

**DOI:** 10.1111/jcmm.13580

**Published:** 2018-03-07

**Authors:** Jieying Chen, Xunan Sheng, Hongchang Ma, Zhengshan Tang, Chao Yang, Lanqin Cao, Yang Sun, Tanggang Deng, Peifu Feng, Bin Hu, Dong Wei, Jing Liu, Wei Xiong, Mao Ye

**Affiliations:** ^1^ Molecular Science and Biomedicine Laboratory State Key Laboratory for Chemo/Biosensing and Chemometrics College of Biology College of Chemistry and Chemical Engineering Collaborative Innovation Center for Molecular Engineering for Theranostics Hunan University Changsha Hunan China; ^2^ College of Life and Environmental Sciences Gannan Normal University Ganzhou Jiangxi China; ^3^ Department of Gynecology Xiangya Hospital Central South University Changsha Hunan China; ^4^ School of Life Sciences Central South University Changsha Hunan China; ^5^ Ophthalmology and Eye Research Center The Second Xiangya Hospital Central South University Changsha Hunan China

**Keywords:** lung cancer, proliferation, stability, ubiquitin

## Abstract

WD repeat protein 79 (WDR79) is a member of the WD‐repeat protein family characterized by the presence of a series of WD‐repeat domains and is a scaffold protein that participates in telomerase assembly, Cajal body formation and DNA double strand break repair. Although previous studies have revealed that WDR79 is frequently overexpressed in non‐small cell lung cancer (NSCLC) and promotes the proliferation of NSCLC cells, the underlying mechanism responsible for WDR79‐mediated NSCLC proliferation is not fully understood. In this study, we report a novel molecular function of WDR79 that mediates NSCLC cell proliferation by controlling the stability of UHRF1. In the nucleus, WDR79 colocalized and interacted with UHRF1. As a result, overexpression of WDR79 stabilized UHRF1, whereas ablation of WDR79 decreased the level of UHRF1. Meanwhile, we showed that WDR79 can protect UHRF1 from poly‐ubiquitination‐mediated proteolysis, which facilitated the stabilization of UHRF1. We further demonstrated that WDR79 exerts a proliferation effect on NSCLC cells by stabilizing UHRF1. These findings reveal that WDR79 is a novel UHRF1 regulator by maintaining UHRF1 stability, and they also provide a clue as to how to explore WDR79 for potential therapeutic application in NSCLC.

## 1 INTRODUCTION

Lung cancer is the disease with the highest morbidity and mortality rate in the world.^1^ In 2017, it has been estimated that lung cancer will account for 13% of all new cancer cases and for 26% of cancer‐related deaths.^2^ Non‐Small Cell Lung Cancer (NSCLC) is the predominant type of lung cancer and accounts for approximately 80%‐85% of all lung cancer cases. Two‐thirds of NSCLC patients exhibit an advanced stage at diagnosis. Despite recent advances in therapeutic strategies, the prognosis for NSCLC patients remains poor with less than 15% of the 5‐year survival rate. Therefore, it is imperative to clarify the molecular mechanism of tumorigenesis for effective manipulation of NSCLC.

WDR79 (also referred to as WRAP53β/TCAB1) is a member of the WD‐repeat proteins family and contains six individual WD‐repeat domains that begin with a glycine‐histidine (GH) dipeptide and end with a tryptophan‐aspartic acid (WD) dipeptide. Functionally, WDR79 serves as a scaffold protein through the β‐propeller platform structure formed by WD‐repeat domains and is involved in telomerase assembly, Cajal body formation and DNA double stand break repair.^3^, ^4^, ^5^, ^6^, ^7^, ^8^ Emerging studies have shown that aberrations in WDR79 expression correlate with many different malignancies, such as rectal cancer,^9^ head and neck carcinomas,^10^ oesophageal squamous cell carcinoma,^11^ breast cancer,^12^ ovarian cancer 13 and nasopharyngeal carcinoma.^14^ Our previous studies revealed that WDR79 is frequently overexpressed in cell lines and tissues derived from non‐small cell lung cancer and promotes the proliferation of NSCLC cells,^15^, ^16^ which is consistent with a recent study.^17^ However, the underlying mechanism responsible for WDR79‐mediated NSCLC proliferation is not fully understood.

Ubiquitin‐like with PHD and RING finger domains 1 (UHRF1) is a protein with a multiple functional domain, which acts as an epigenetic coordinator by regulating replication‐coupled crosstalk between DNA methylation and histone modifications.^18^ UHRF1 binds hemimethylated DNA via its SET‐ and RING‐associated (SRA) domain and recruits DNA methyltransferase 1 (DNMT1) to methylate the newly synthesized strand. Meanwhile, UHRF1 also serves as a E3 ubiquitin‐protein ligase to promote ubiquitylation of histone H3 by its RING domain, which provides the docking site for DNMT1.^19^ Studies have shown that the UHRF1 protein is regulated at both transcriptional and post‐translational levels. Ubiquitylation is one of the most important reversible post‐translational modifications of UHRF1. It is well‐known that UHRF1 can be ubiquitylated and degraded by the SCF^β−TrCP^ E3 ubiquitin ligase complex.^20^ On the other hand, the ubiquitin‐specific‐processing protease 7 (USP7) removes ubiquitin conjugated to UHRF1 and prevents proteasomal degradation of UHRF1.^21^, ^22^

UHRF1 is mainly expressed in proliferating cells and tissues but not in highly differentiated tissues.^23^, ^24^ High expression of UHRF1 has frequently been found in a variety of human cancers. Previous studies reveal that UHRF1 is overexpressed in almost all histological types of lung cancer and correlates with a poor prognosis, which can be useful for diagnosis of lung cancer in all pathological stages.^25^ In NSCLC, UHRF1 overexpression resulted in the silencing of tumour suppressor genes by maintaining their promoters in a hypermethylated state.^26^

In this study, we identified UHRF1 as a unique WDR79 interacting protein. WDR79 positively regulates UHRF1 stability by protecting it from ubiquitin‐mediated degradation, and this positive regulation of UHRF1 by WDR79 mediates the proliferation of NSCLC.

## 2 MATERIALS AND METHODS

### 2.1 Cell culture and transfection

H1299 and A549 cells cell lines were purchased from the Shanghai Cell Bank of the Chinese Academy of Sciences (Shanghai, China) and cultured in RPMI‐1640 medium (Gibco BRL Co. Ltd., Grand Island, NY, USA) with 10% foetal bovine serum (Gibco BRL Co. Ltd.). Transfections with various expression plasmids were performed using HD FuGENE reagents (Roche), according to the suggested protocol by the manufacturer.

### 2.2 Plasmids and antibodies

WDR79 or UHRF1, the full‐length mRNA sequences, were PCR‐amplified from human cDNA and subcloned into pCMV‐Tag2B to create Flag‐tagged WDR79 expression plasmids. The WDR79 and UHRF1 antibodies were from Bethyl Laboratories (Montgomer y, TX, USA), the GAPDH antibody was obtained from KangChen Bio‐tech Inc (Shanghai, China) and the antibody against ubiquitin (6C1.17) was from BD Pharmingen (Franklin Lakes, NJ, USA).

### 2.3 RNA interference

The sequence of the UHRF1 siRNA was 5′‐GCUCAUGUGCGAUGAGUGC‐3′, and the sequence of the control siRNA was 5′‐UUCUCCGAACGUGUCACGUTT‐3′ (all synthesized by Shanghai GenePharma, China). siRNA transfection was performed according to the manufacturer's protocol. To stably knock down endogenous WDR79 in some case, we used lentivirus‐packaging shRNA expression vector (purchased from Shanghai GenePharma, China) to infect cells. WDR79 shRNA target sequence was 5′‐AATCAGCGCATCTACTTCGAT‐3′, and the control shRNA sequence was 5′‐TTCTCCGAACGTGTCACGTTTC ‐3′.

### 2.4 Western blotting assay

Cells were harvested and lysed with M‐PER buffer containing protease inhibitors (Pierce, Rockford, IL, USA). Proteins were resolved on 10% SDS‐PAGE gels and transferred to nitrocellulose‐membranes. Then, membranes were blocked with 5% non‐fat milk for 1 hour at room temperature and incubated with primary antibodies overnight at 4°C. Thereafter, membranes were washed and incubated with appropriate horseradish peroxidase‐conjugated secondary antibodies (Santa Cruz). The results were obtained using a chemiluminescence detection kit (Pierce).

### 2.5 Immunoprecipitation assay

Immunoprecipitations were performed using the Universal Magnetic Co‐IP Kit (Active Motif, Carlsbad, CA, USA). First, 1 mg of crude extract was incubated with 3 μg of a relevant primary antibody or an isotype‐matched negative control IgG overnight at 4°C. Second, samples were incubated with 30 μL of magnetic beads conjugated with an antibody for 1 hour at room temperature and washed three times with Co‐IP/wash buffer. The immune complex was captured by Protein A or Protein G agarose beads by incubation for 1 hour. Any non‐bound protein (non‐immune complex sample components) was removed from the precipitated complex by washing beads with Co‐IP/wash buffer. Next, precipitated proteins were dissolved in 30 μL 2× SDS protein loading buffer, boiled for 10 minutes at 95°C and subjected to Western blotting analysis.

### 2.6 Indirect immunofluorescence assay

H1299 cells were washed three times with DPBS and fixed with 4% paraformaldehyde (PFA) for 20 minutes, then washed three times with DPBS and permeabilized with 0.2% Triton X‐100 in DPBS for 15 minutes, and blocked with 5% BSA (bovine serum albumin) for 1 hour. Cells were then incubated with anti‐WDR79 (Bethyl Laboratories, Inc., Montgomery, TX, USA) or anti‐UHRF1 (Bethyl Laboratories, Inc., Montgomery, TX, USA) antibodies at 4°C overnight followed by a DyLight 594‐conjugated or DyLight 488‐conjugated secondary antibody (ImmunoReagents, Inc., Raleigh, NC, USA). Cells were stained with 2‐(4‐Amidinophenyl)‐6‐indolecarbamidinedihydrochloride (DAPI) (Beyotime Biotechnology, Haimen, China) for 10 minutes and washed three times with PBST; the images were acquired with a confocal microscope.

### 2.7 Protein half‐life assay

H1299 Cells were treated with cycloheximide (50 μg/mL) for various periods of time to block protein synthesis. Cells were harvested at specified time‐points. Crude extracts were prepared, and protein levels were assessed by Western blotting analysis.

### 2.8 In vivo ubiquitination assay

First, cells were incubated with 20 μmol/L MG132 for 6 hours. Then, cells were lysed and incubated with anti‐UHRF1 antibodies overnight at 4°C. Last, 30 μL of beads conjugated with protein G was added and incubated for 1 hour at room temperature. The immunoprecipitates were dissolved in 40 μL of 2× SDS protein loading buffer and tested with Western blotting using an anti‐ubiquitin antibody**.**

### 2.9 Cell proliferation assay

Cells were seeded in 96‐well culture plates; 3–(4,5‐dimethylthiazol‐2‐yl)‐2,5‐diphenyltetrazolium bromide (Sigma, St. Louis, MO, USA) solution was added to each well at the indicated time‐point and incubated at 37°C for another 4 hours. Supernatants were then aspirated, and the formazan product was dissolved with 100 μL dimethyl sulfoxide. The absorbance was measured at a wavelength of 570 nm with a microplate reader (Bio‐Tek, Doraville, GA, USA).

## 3 RESULTS

### 3.1 WDR79 influences the protein level of UHRF1

**Figure 1:**
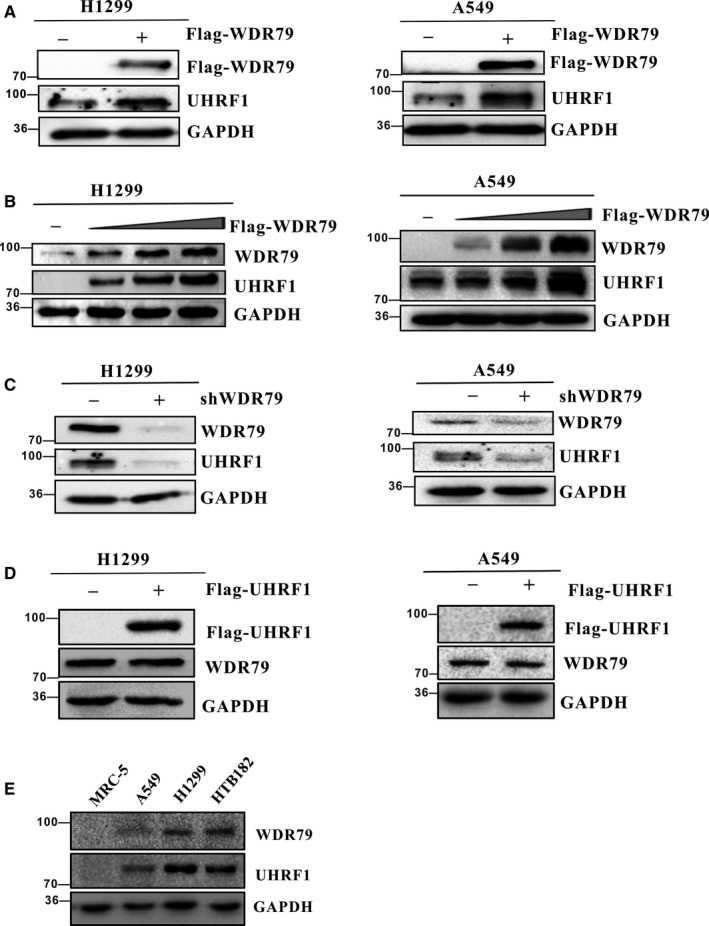
WDR79 influence on the level of UHRF1. (A) H1299 and A549 cells were transfected with a plasmid encoding Flag‐WDR79 or an empty vector control. Total protein was extracted and subjected to Western blotting using the indicated antibodies. (B) Increasing amounts of Flag‐WDR79 were transfected into H1299 and A549 cells, and total protein was extracted from these cells and subjected to Western blotting using the indicated antibodies. (C) H1299 and A549 cells were infected with the indicated lentivirus shRNAs. The resulting cell extracts were analysed using Western blotting with the indicated antibodies. (D) H1299 and A549 cells were transfected with a plasmid encoding Flag‐UHRF1 or an empty vector control. Total protein was extracted and subjected to Western blotting using the indicated antibodies. (E) The expression level of WDR79 and UHRF1 in MRC‐5, A549, H1299 and HT182 cells was examined by Western blotting using WDR79 and UHRF1 antibody

**Figure 2:**
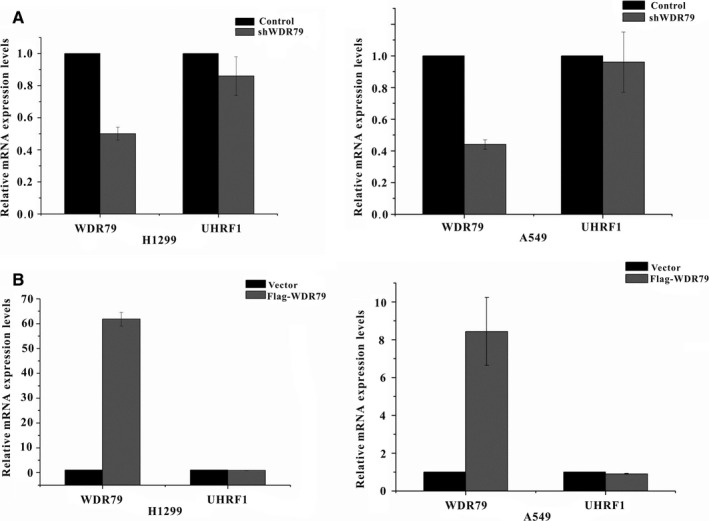
WDR79 did not change UHRF1 mRNA levels. (A) H1299 and A549 cells were transfected with a plasmid encoding Flag‐STIP or an empty vector control, and the relative mRNA expression levels of WDR79 and UHRF1 were measured by real‐time PCR. (B) H1299 and A549 cells were infected with lentivirus encoding the WDR79 shRNA or control shRNA, and relative mRNA expression levels of WDR79 and UHRF1 were measured by real‐time PCR

Compelling evidence suggests a strong link between UHRF1 overexpression and NSCLC. To clarify the potential mechanism by which WDR79 regulates the proliferation and tumorigenesis of NSCLC, we investigated whether WDR79 affected the level of UHRF1. WDR79 overexpression was performed, and its effect on the level of endogenous UHRF1 was assayed in A549 and H1299 cells. Intriguingly, WDR79 overexpression significantly increased the level of UHRF1 compared with the vector control (Figure 1A), and a gradual increase in the expression level of WDR79 caused an elevation in UHRF1 levels in a dose‐dependent manner in all cell lines (Figure 1B). To further confirm the effect of WDR79 on UHRF1, we then performed a loss‐of‐function analysis using WDR79‐specific short hairpin RNAs (shRNAs) in A549 and H1299 cells. As expected, WDR79 knockdown dramatically decreased the level of UHRF1 (Figure 1C). In contrast, overexpression of UHRF1 did not change WDR79 levels in H1299 and A549 cells (Figure 1D). To further determine whether there was a correlation between WDR79 and UHRF1, we detected the expression of endogenous WDR79 and UHRF1 in human lung fibroblasts MRC‐5 and human lung cancer cell lines A549, H1299 and HTB182. Interestingly, the absence of WDR79 expression was accompanied with the loss of UHRF1 expression in MRC‐5 cells, whereas a positive correlation between WDR79 and UHRF1 was found in tested lung cancer cell lines (Figure 1E). Meanwhile, we employed real‐time polymerase chain reaction (PCR) to assess UHRF1 mRNA expression after overexpression or down‐regulation of WDR79. We found that the levels of UHRF1 mRNA were not significantly affected by WDR79 (Figure 2A,B). Thus, WDR79 regulates UHRF1 at the protein level but not at the transcriptional level.

### 3.2 WDR79 regulates UHRF1 protein levels through the proteasome pathway

**Figure 3:**
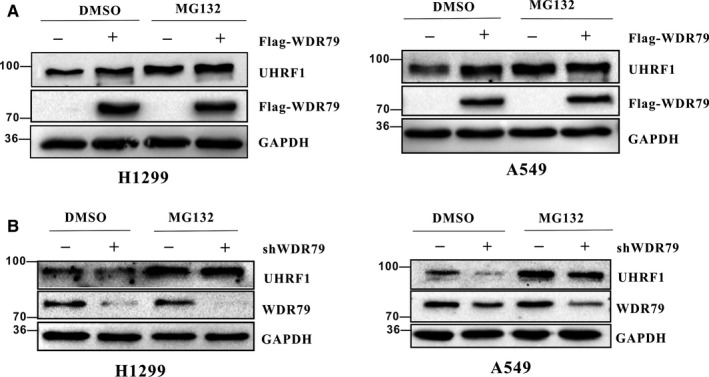
WDR79 regulates UHRF1 protein levels through the proteasome pathway. (A) H1299 and A549 cells transfected with Flag‐UHRF1 plasmid or control plasmid were incubated with 20 μmol/L MG132 for 6 h. Expression of the indicated proteins was examined by Western blotting using the indicated antibodies. (B) H1299 and A549 cells infected with lentivirus encoding the WDR79 shRNA or control shRNA were incubated with 20 μmol/L MG132 for 6 h. Expression of the indicated proteins was examined by Western blotting using the indicated antibodies

It is well‐known that approximately 80% of intracellular protein is degraded via the proteasome pathway. As WDR79 interacts with UHRF1 and regulates UHRF1 protein levels, we surmised that WDR79 mediates the regulation of UHRF1 protein levels through the proteasome pathway. To test this possibility, A549 and H1299 cells transfected with vector and Flag‐WDR79 were treated with the proteasome inhibitor MG132, and the level of endogenous UHRF1 protein was examined by Western blotting. As expected, in the absence of MG132, the overexpression of WDR79 resulted in a significant increase in endogenous UHRF1 levels. However, MG132 treatment completely abolished the change caused by WDR79 (Figure 3A). Similar results were also obtained in A549 and H1299 cells with WDR79 knockdown using WDR79‐specific shRNA (Figure 3B). Collectively, these results demonstrate that WDR79 regulates UHRF1 levels in a proteasome‐dependent manner.

### 3.3 WDR79 interacts with UHRF1

**Figure 4:**
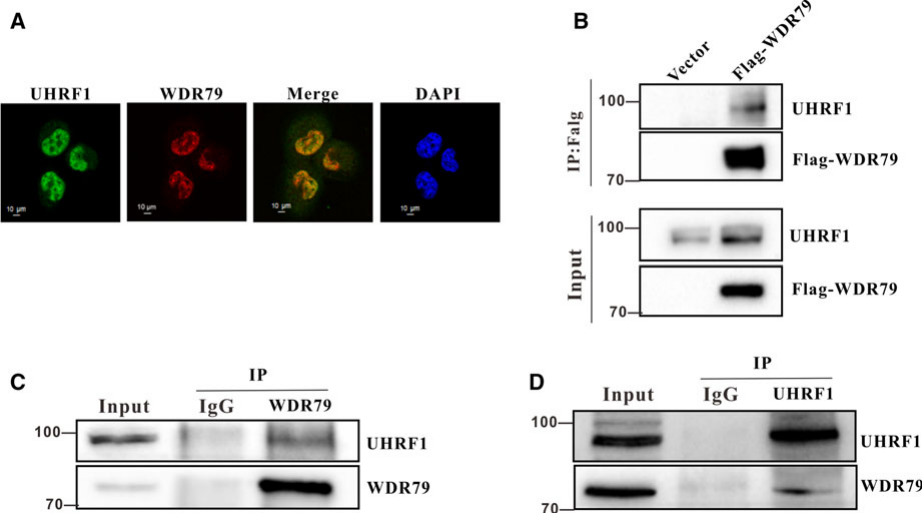
WDR79 interacts with UHRF1. (A) The subcellular localization of endogenous WDR79 (green) and UHRF1 (red) in H1299 cells were visualized using immunofluorescence with anti‐WDR79 and UHRF1 antibodies. DNA was stained with DAPI, and a merged view of the red and green channels within the same field is shown (merge). (B) H1299 cells were transfected with a Flag‐WDR79 plasmid or an empty vector control plasmid. An anti‐flag antibody was used for immunoprecipitation, and immunoprecipitates were analysed by Western blotting using the indicated antibodies. (C and D) H1299 cell lysates were precipitated with anti‐WDR79 (C), anti‐UHRF1 (D) or control IgG antibodies. The immunoprecipitates were then probed with the indicated antibodies

To understand how WDR79 regulates UHRF1 levels, we examined whether WDR79 interacts with UHRF1. Endogenous WDR79 and UHRF1 in H1299 cells were labelled with their corresponding antibodies conjugated to Dylight 594 (red) and Dylight 488 (green), respectively. Consistent with previous reports, the subcellular localizations of WDR79 and UHRF1 were in the nucleus. In the merged images, an intense yellow colour indicated colocalization of WDR79 and UHRF1 (Figure 4A). To confirm that WDR79 and UHRF1 indeed interact with each other in vivo, Flag‐WDR79 plasmid was transfected into H1299 cells. The expression of Flag‐WDR79 and its interaction with endogenous UHRF1 were then investigated by coimmunoprecipitation (co‐IP) and Western blotting (WB) analysis. As shown in Figure 4B, UHRF1 was co‐immunoprecipitated by an anti‐Flag antibody in WDR79‐overexpressing cells but not in negative control cells transfected with the same amount of empty vector. Furthermore, the endogenous interaction between WDR79 and UHRF1 was also detected. WDR79 and UHRF1 were separately immunoprecipitated from H1299 cells, and reciprocal protein detection was performed. As shown in Figure 4C,D, both WDR79 and UHRF1 were detectable in their individual immunoprecipitated complexes but not in an isotype‐matched negative control IgG. Taken together, these data indicate that WDR79 and UHRF1 interact with each other.

### 3.4 WDR79 affects the half‐life of UHRF1 protein

**Figure 5:**
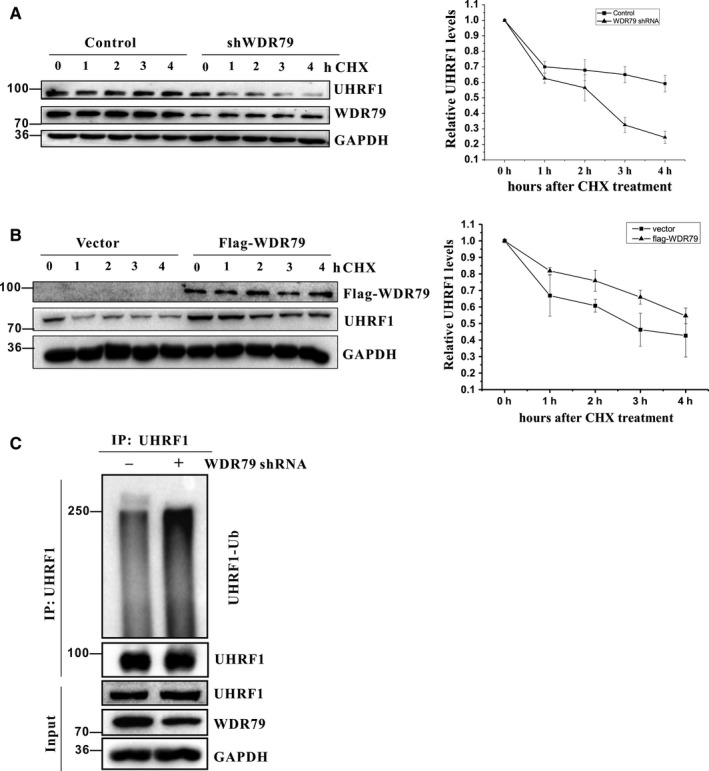
WDR79 affects the half‐life of UHRF1. (A) H1299 cells infected with a lentivirus encoding the WDR79 shRNA or control shRNA were treated with 50 μg/mL cycloheximide (CHX), collected at the indicated time points and immunoblotted with the indicated antibodies. Quantification of the UHRF1 levels relative to GAPDH expression was shown. (B) H1299 cells transfected with the indicated constructs were treated with 50 μg/mL cycloheximide (CHX), collected at the indicated time‐points and immunoblotted with the indicated antibodies. Quantification of the UHRF1 levels relative to GAPDH expression is shown. (C) H1299 cells infected with a lentivirus encoding the WDR79 shRNA or control shRNA were incubated with 20 μmol/L MG132 for 6 h. Lysates were immunoprecipitated with anti‐UHRF1. The ubiquitylation of UHRF1 was analysed by Western blotting using an anti‐ubiquitylation antibody

Given that WDR79 regulates the protein level of UHRF1, we hypothesized that WDR79 may affect UHRF1 protein stability. To explore this hypothesis, H1299 cells with or without WDR79 knockdown were treated with cycloheximide (CHX) to inhibit protein biosynthesis, and protein extracts obtained at different time points were analysed. As shown in Figure 5A, the half‐life of UHRF1 in WDR79‐depleted cells was significantly shorter than that in control cells. In contrast, ectopically expressed WDR79 profoundly extended the half‐life of UHRF1 protein (Figure 5B). Thus, WDR79 is essential for the maintenance of the stability of UHRF1. To further determine the underlying mechanism whereby WDR79 regulates the stability of UHRF1, we measured the levels of polyubiquitination of UHRF1 in H1299 cells. As expected, WDR79 knockdown resulted in a significant increase in UHRF1 polyubiquitination (Figure 5B), suggesting that WDR79 controls the stability of UHRF1 by modulating its ubiquitination for proteasome degradation.

### 3.5 WDR79 promotes the proliferation of NSCLC cells through UHRF1

**Figure 6:**
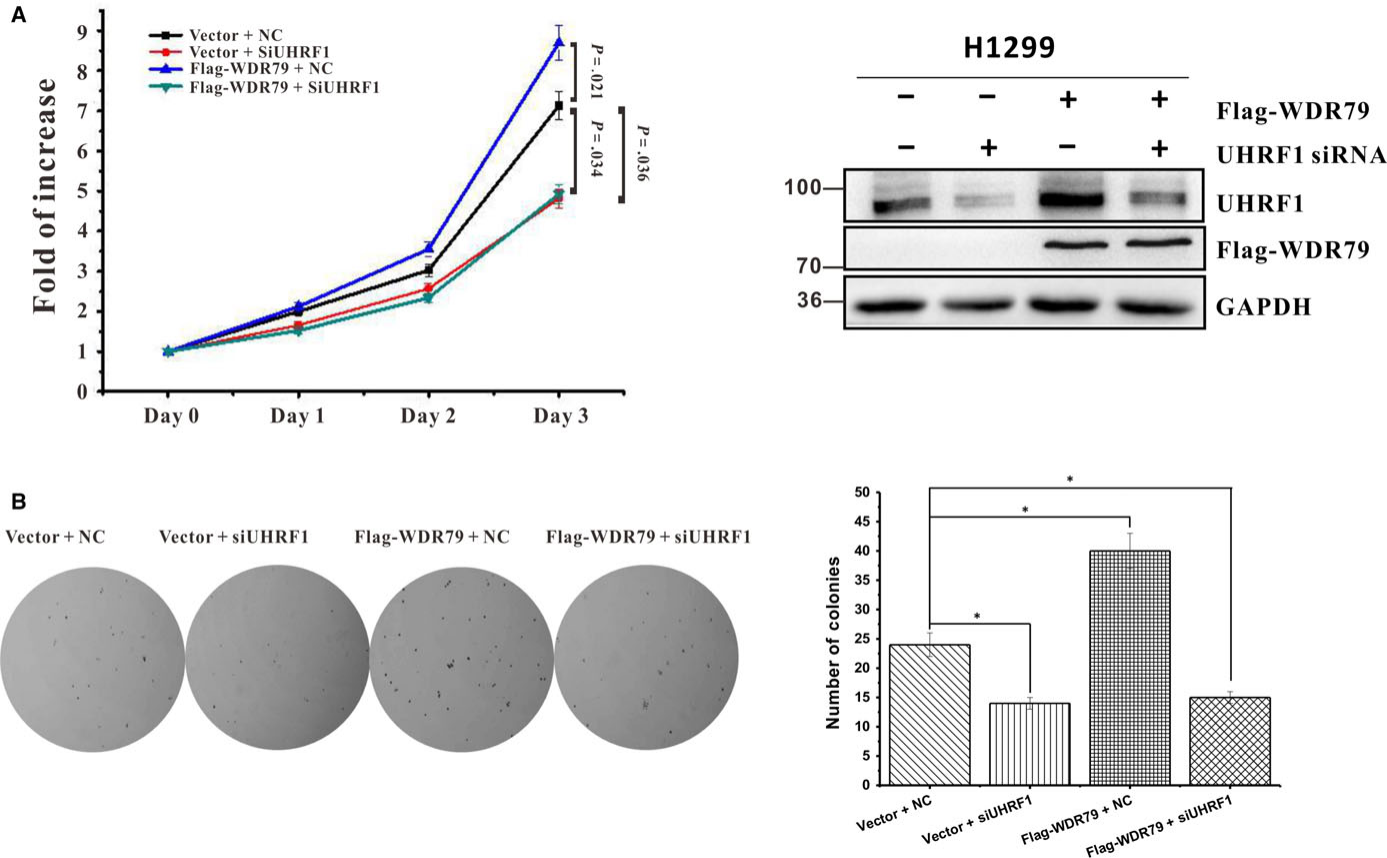
WDR79 promotes NSCLC cell proliferation through UHRF1. (A and B) H1299 cells with or without UHRF1 knockdown were transfected with Flag‐WDR79 or vector. Cell proliferation was analysed with MTT assay at the indicated time‐points (A) or Soft agar colony‐formation assay was performed (B). The error bars represent the mean ± SD of three independent experiments. *P < .05

In previous studies, we found that WDR79 was frequently overexpressed in cell lines and tissues derived from NSCLC, and it enhanced the proliferation of NSCLC cells both in vitro and in vivo.^15^, ^16^ Functionally, WDR79 served as a scaffolding protein that helped to coordinate the various functions of cellular proteins. Given that UHRF1 is closely related to NSCLC, we next asked whether WDR79 mediated the proliferation of NSCLC cells via UHRF1. To this end, we used siRNA to knock down the expression of UHRF1, followed by overexpression of WDR79 in H1299 cells. In agreement with previous reports, UHRF1 knockdown inhibited the proliferation of H1299 cells, whereas WDR79 overexpression accelerated cell proliferation compared with control cells. However, UHRF1 down‐regulation effectively abolished the proliferation‐promoting effect of WDR79 on H1299 cells (Figure 6A). Similar results were obtained with soft agar colony formation assay (Figure 6B), indicating that WDR79‐mediated cell proliferation depends on UHRF1.

## 4 DISCUSSION

WD‐repeat proteins are characterized by the presence of a series of four to more repeating units that contain a conserved core of 40‐60 amino acids that begin with a glycine‐histidine (GH) dipeptide and end with a tryptophan‐aspartic acid (WD) dipeptide.^27^ In general, WD‐repeat proteins serve as a scaffold for protein interactions in various cellular events.^28^ WDR79 is a member of the WD‐repeat protein family, which contains six WD‐repeat domains. In this study, we identified that WDR79 interacted with UHRF1 in the nucleus. Overexpression of WDR79 stabilized UHRF1, whereas WDR79 depletion led to the destabilization of UHRF1, which was accompanied by increased ubiquitination. Functionally, WDR79 mediated the proliferation of NSCLC through UHRF1. Thus, our results provide a new insight into the molecular function of WDR79 in NSCLC.

The ubiquitin‐proteasome system mediated by E3 ubiquitin ligases and deubiquitylases is one of the key ways to regulate the stability and activity of intracellular proteins.^29^ Numerous studies show that WD‐repeat proteins are involved in the ubiquitin‐proteasome process by promoting multiple protein complex assembly. WDR23 along with the ubiquitin ligase CUL4/DDB1 function to trigger the degradation of the SKN‐1 transcription factor,^30^ whereas WDR48 contacts USP46 via a β‐propeller platform structure formed by WD‐repeat domains and stimulates its activity to stabilize substrates.^31^ We have also previously demonstrated that WDR79 interacts with and regulates USP7 function to determine the stability of p53 and Mdm2.^15^ In the present work, we further found that WDR79 can bind to and stabilize UHRF1. Although studies have shown that USP7 can stabilize UHRF1, we failed to detect that WDR79 coordinated USP7 function on UHRF1. Given that WDR79 does not possess any enzymatic activity, we speculate that WDR79 may regulate UHRF1 stability by another yet unidentified deubiquitylase. Further studies are needed to fully understand the regulation of UHRF1 mediated by WDR79.

UHRF1 functions as an epigenetic hub protein that facilitates crosstalk between DNA methylation and histone modification in the cell.^32^ Through its SET‐ and RING‐associated (SRA) domain, UHRF1 recognizes hemimethylated DNA and induces the recruitment of DNA methyltransferase 1 (DNMT1) to methylate the newly synthesized strand. Meanwhile, UHRF1 ubiquitylates histone H3 by its RING domain to provide the docking site for DNMT1. Currently, although there is no evidence that WDR79 is involved in an epigenetic process, WDR79‐mediated UHRF1 stability will provide a clue for further investigation.

UHRF1 has been shown to be overexpressed in various types of lung cancer, which can be useful for diagnosis of lung cancer in all pathological stages.^25^ Through maintaining the promoters of tumour suppressor genes in a hypermethylated state, UHRF1 inhibits their expression at a transcription level and promotes the proliferation of NSCLC.^26^ Consistent with the expression pattern of UHRF1, we found that WDR79 was overexpressed in NSCLC. Ectopically expressed WDR79 profoundly extended the half‐life of UHRF1, whereas down‐regulation of WDR79 decreases levels of UHRF1. This implied that increased expression of UHRF1 in NSCLC depended on WDR79. Thus, our study will contribute to the identification of novel strategies to manipulate WDR79 function for future therapeutic applications in NSCLC.

## CONFLICT OF INTERESTS

The authors declare that no competing financial interests exist.
